# Comparison of the IPSA and HIPO algorithms for interstitial tongue high-dose-rate brachytherapy

**DOI:** 10.1371/journal.pone.0205229

**Published:** 2018-10-04

**Authors:** Chang Heon Choi, So-Yeon Park, Jong Min Park, Hong-Gyun Wu, Jin-Ho Kim, Jung-in Kim

**Affiliations:** 1 Department of Radiation Oncology, Seoul National University Hospital, Seoul, Republic of Korea; 2 Biomedical Research Institute, Seoul National University College of Medicine, Seoul, Republic of Korea; 3 Institute of Radiation Medicine, Seoul National University Medical Research Center, Seoul, Republic of Korea; 4 Center for Convergence Research on Robotics, Advanced Institutes of Convergence Technology, Suwon, Republic of Korea; 5 Department of Radiation Oncology, Seoul National University College of Medicine, Seoul, Republic of Korea; Roswell Park Cancer Institute, UNITED STATES

## Abstract

**Purpose:**

This study aimed to compare the inverse planning simulated annealing (IPSA) stochastic algorithm with the hybrid inverse planning and optimization (HIPO) algorithm for interstitial tongue high-dose-rate (HDR) brachytherapy.

**Methods:**

Twenty patients who received radiotherapy for tongue cancer using interstitial HDR brachytherapy were retrospectively selected for this study. Oncentra Brachy v. 4.3 was used for IPSA and HIPO planning. Four to eight fixed catheter configurations were determined according to the target shape. During the optimization process, predetermined constrain values were used for each IPSA and HIPO plan. The dosimetric parameters and dwell time were analyzed to evaluate the performances of the plans.

**Results:**

The total dwell time using IPSA was 4 seconds longer than that of HIPO. The number of active positions per catheter for the IPSA plans were approximately 2.5 fewer than those of the HIPO plans. The dose-volumetric parameters related to the clinical target volume with IPSA were lower than those with HIPO. In terms of the dose-volumetric parameters related to normal tissue, HIPO tended to associate with slightly higher values than IPSA, without statistical significance. After GrO, the target coverages were satisfied to clinical goal for all patients. The total dwell times was approximately increased by 10%.

**Conclusions:**

The IPSA and HIPO dose optimization algorithms generate similar dosimetric results. In terms of the dwell time, HIPO appears to be more beneficial.

## Introduction

High-dose-rate (HDR) brachytherapy has been established as an effective treatment method for early-stage tongue cancer [1–3]. Moreover, brachytherapy results in better functional and cosmetic results than surgery, with similar tumor control [4–6]. Brachytherapy provides a high localized dose of radiation to the tumor and reduces the volume of irradiated normal tissues, with rapid dose fall-off [7–9]. Consequently, the tumor control can be improved and the probability of side effects (i.e., xerostomia and soft tissue fibrosis) can be reduced [9–12].

For the treatment planning process, computed tomography (CT) is performed to obtain anatomical information and to determine the appropriate catheter implantation location. Treatment planning is performed by optimizing the source dwell positions and dwell times needed to cover the target with the prescription dose while reducing the dose to the normal tissues based on CT imaging [13], thereby enabling the generation of more accurate treatment plans. Depending on the tumor volume, 5–10 catheters are inserted, with potential dwell positions every 25 mm. Accordingly, iterative forward planning is a time-consuming and laborious process, owing to the numerous different combinations of dwell times and positions for optimal planning [14].

As an alternative to forward planning, inverse planning optimization for HDR brachytherapy planning has been reported [15, 16]. During the inverse planning process, the optimized plan is generated to have a high target coverage, low dose to normal tissues, and optimal dose distribution based on dose constraints contoured according to anatomic information [15]. As optimizers, the Inverse Planning Simulated Annealing (IPSA) optimization algorithm and Hybrid Inverse Planning and Optimization (HIPO) algorithm have been implemented using the commercially available Oncentra Brachytherapy treatment planning system v. 4.3 (Nucletron B.V., Veenendaal, The Netherlands) [17].

IPSA is a CT-based inverse planning method that can produce an optimized plan within 1 minute [18]. IPSA has been widely used for clinical purposes and has been demonstrated to have clinical efficiency. However, stochastic algorithms are known to be slow, and the dwell times have an inhomogeneous distribution [19, 20]; some dwell positions have dominating dwell times while others have short or zero dwell times [21]. These varying distributions of dwell positions can potentially produce heterogeneous dose distributions [22].

As an alternative to IPSA, HIPO has been introduced in HDR brachytherapy [23]. HIPO is another inverse planning algorithm based on 3D anatomical information. HIPO can optimize the dose distribution for pre-implant catheter configurations [24] and can be used to determine the feasible positions of catheters. Moreover, HIPO can reduce the selective hot spots to provide more uniform dwell time distribution, thereby minimizing the free dwell times of adjacent dwell positions [23].

The clinical superiority of anatomy-based inverse treatment plan optimizations (i.e., IPSA and HIPO) for HDR brachytherapy of prostate and cervical cancers has been demonstrated by several groups [18, 19, 21, 25–27]. However, although a role of HDR brachytherapy has been demonstrated in tongue cancer treatment, the clinical effectiveness of inverse planning has not been evaluated for tongue cancer.

In this work, we investigated the plans generated using HIPO vs. IPSA for tongue cancer patients. Specifically, the clinically relevant dose–volumetric and plan parameters (i.e., dwell position and time) were analyzed to evaluate the quality of these two algorithms. After each optimization, if target coverage is not satisfied to clinical goal, graphical optimization (GrO) was performed to accomplish an acceptable coverage.

## Materials and methods

### Patient selection and simulation

The tongue cancer patients of 20 treated by only HDR in our institution were randomly enrolled in this study. It was approved by the institutional review board of Seoul National University College of Medicine/Seoul National University Hospital (IRB No.1611-078-809) for this study. In this retrospective study, all of patient’s DICOM-RT set was anonymized and minimal risk cause to the patient, the IRB approved exemption from informed consent for this study. All patients underwent CT scans using the Brilliance CT Big Bore (Philips, Amsterdam, Netherlands), with a slice thickness of 3.0 mm. The clinical target volume (CTV) was defined at the time of planning by one oncologist. The mandible and normal tongue were contoured by one dosimetrist. For planning, the tongue_only structure was generated by extracting the CTV from the normal tongue. The prescription dose was 45 Gy in 9 fractions.

The mean CTV was 7.69 ± 2.50 cm^3^ (range, 5.91–15.09 cm^3^), and the volumes of the mandible and tongue were 81.43 ± 11.86 cm^3^ and 61.50 ± 15.02 cm^3^, respectively.

Treatment planning was performed using the Oncentra Brachytherapy treatment planning system v. 4.3 (Nucletron B.V.) with a ^192^Ir (mHDR-v2r) source for the HDR unit (microSelectron v3). At the time of planning, the air-kerma strength was 48950.0 cGy cm^2.^h^-1^ and the apparent source activity was 11.99 Ci.

For interstitial brachytherapy, the Paris system has been recommended to determine the placement of the catheter. The sources should be straight, parallel, and of equal length, and the catheters should have equal separation between the sources [28]. However, this system cannot be applied to tongue cancer since the shape of the target is irregular and the volume is small. Therefore, the number and placement of the catheters were determined clinically in consideration of the target size and shape. The catheters were placed 1 cm apart throughout the target, at least 5 mm away from the border of the target. Depending on the tumor size and shape, 4–8 catheters were reconstructed. The source position separation was set to 2.5 mm.

### IPSA optimization

First, plan optimization was performed with the IPSA algorithm. Table 1 shows the initial parameters of IPSA. To assess the initial dose value and weight of normal tissues (i.e., mandible and tongue) during the optimization process, the optimization process was performed repeatedly by changing the initial constraint. The maximum values were determined as the constraints of normal tissue within the range to ensure 90% coverage of the CTV with 100% of the prescription for all patients. The dwell time deviation constraint parameter was set to zero since it was not used clinically in our institution, and the effect of the dwell time deviation constraint was hence not established.

**Table 1:** Inverse planning simulated annealing (IPSA) optimization algorithm parameters.

					Surface			Volume		
	Margin				Min	Max		Min	Max	
ROI	Usage	(cm)	Actv.	Weight	(Gy)	(Gy)	Weight	(Gy)	(Gy)	Weight
CTV	Target	0.5	0.5	100	5	15	100	5	10	50
Tongue	Organ	0	0	50		5	50			
Mandible	Organ	0	0	80		3.5	80			

Abbreviations: ROI region of interest, CTV clinical target volume

### HIPO optimization

The HIPO algorithm uses a stochastic model to optimize the catheter distribution (heuristic algorithm) and a deterministic model to perform the inverse optimization of the dwell time for the defined catheter configuration (quasi-Newton algorithm) [17].

In the case of prostate interstitial brachytherapy, HIPO selects the catheter positions using template holes projected on the reference image. The catheter positions are optimized by the iterative method based on contoured structures on the reference image and the margins [19]. However, it is very difficult to use template holes for tongue cancer considering the target shape. Therefore, in this study, the catheter positions used for treatment planning were the same as those manually determined for IPSA planning. HIPO was only performed for dose optimization. Table 2 shows the dosimetric constraints of HIPO. HIPO can adjust the priorities of the target and the organs at risk (OARs) during the optimization. For each patient, three plans were generated by assigning priorities of 1–3 to the different structures. The priorities of the PTV, mandible, and tongue were assigned as 1 for the HIPO_CTV, HIPO_OAR_Man, and HIPO_OAR_Ton, respectively. The last three columns in Table 2 show the priority combinations in this study.

**Table 2:** Hybrid inverse planning and optimization (HIPO) algorithm optimization parameters.

			Min	Max		Priority		
ROI	Usage	Min. weight	(Gy)	(Gy)	Max Weight	HIPO_CTV	HIPO_OAR_Man	HIPO_OAR_Ton
CTV	Target	100	5	20	100	1	3	3
Tongue	Organ			5	30	3	2	1
Mandible	Organ			3.5	80	2	1	2

Abbreviations: ROI region of interest, CTV clinical target volume, OAR organ at risk, Man mandible, Ton tongue

Further, HIPO provides a dwell time gradient restriction (DTGR) option, which is a modulation restriction parameter to adjust the free modulation of the dwell times. This option allows smoother source movements and more smooth distributions of the dwell time per dwell position in the catheter. However, the value of the DTGR was set as 0 (i.e., this dwell time gradient objective was ignored during optimization) since the DTGR is not a key factor to improve plan quality [18].

### Evaluation of the treatment plans

For IPSA, the HIPO_CTV, HIPO_OAR_Man, HIPO_OAR_Ton, and dosimetric parameters were evaluated. All of the plans were normalized to cover 90% of the CTV with 100% of the prescription dose. For the CTV, the minimum dose to 95% of the target volume (D_95%_) and the volumes receiving 150% and 200% of the prescription (V_150%_ and V_200%_ were calculated. The conformity index (CI) and homogeneity were calculated as follows [29]:

$$\text{Conformity index}\;(\text{CI})=\frac{Volume\;receiving\;prescription\;dose}{Volume\;of\;the\;target\;volume}$$ (1)

For the mandible, the volume irradiated by at least 2 Gy of the dose (V_2Gy_) was calculated for each plan. The mean dose to the tongue was also calculated. The statistical significance of the differences in the dose–volumetric parameters between each plan was analyzed using the paired t-test.

### Graphical optimization (GrO)

The GrO was performed to evaluate the effect of manual isodose line editing. After IPSA and HIPO optimizations, the CTV coverage was evaluated without normalization. If the prescription dose did not cover the 90% of CTV at least, isodose lines were manually adjusted using GrO. The prescription isodose was conformed optimally to the delineated line of CTV. Until acceptable goal (covering at least 90% of the CTV) of plan was achieved, GrO was performed iteratively. The plans modified by GrO were compared to normalized plans.

## Results

Table 3 shows the number of catheter channels and the total dwell time of each plan for each patient. Table 4 show the standard deviation of dwell time per catheter channel. The mean total dwell time of the IPSA plan was longer than that of the HIPO plans (HIPO_CTV, HIPO_OAR_Ton, and HIPO_OAR_Man). The mean difference in the total dwell time was 7.8%. The mean standard deviation (SD) of the total dwell time between the HIPO plans was 0.7s; the maximum SD was 2.7s. The total dwell time using IPSA was significantly longer than that of the HIPO plans (*p* < 0.001), while the total dwell time was similar among the HIPO plans.

**Table 3:** Number of catheters and total dwell time for each plan.

Patient number	Number of catheters	Total dwell time			
		IPSA	HIPO_CTV	HIPO_OAR_Ton	HIPO_OAR_Man
**1**	8	54.9	56.2	56.5	56.3
**2**	5	44.1	43.6	42.9	43.2
**3**	6	59.7	54.3	53.8	53.7
**4**	4	53.7	53.6	52.1	52.3
**5**	6	69.7	69.4	67.8	67.6
**6**	6	62.5	52.2	55.5	55.2
**7**	6	53.3	50.6	49.9	50.1
**8**	6	48.1	38.2	41.7	42.0
**9**	5	48.2	46.1	44.3	44.4
**10**	6	54.2	41.1	41.1	41.0
**11**	4	64.2	63.5	63.8	63.8
**12**	8	55.9	52.6	51.8	51.8
**13**	5	47.6	45.9	46.3	45.8
**14**	8	63.9	61.1	60.0	60.1
**15**	5	48.7	42.7	42.3	42.4
**16**	7	57.6	57.4	56.3	56.3
**17**	6	44.9	44.9	41.1	46.4
**18**	7	48.2	42.0	40.6	41.0
**19**	6	49.0	41.9	41.4	41.7
**20**	8	52.0	43.5	43.4	42.1

Abbreviations: IPSA inverse planning simulated annealing, HIPO hybrid inverse planning and optimization, CTV clinical target volume, OAR organ at risk, Man mandible, Ton tongue

**Table 4:** Standard deviation of the dwell time per catheter channel.

Patient number	Standard deviation of the dwell time per catheter channel			
	IPSA	HIPO_CTV	HIPO_OAR_Ton	HIPO_OAR_Man
**1**	9.2	4.9	5.1	5.2
**2**	3.8	2.1	2.1	2.0
**3**	6.3	2.1	2.2	2.2
**4**	5.1	3.7	3.7	3.7
**5**	3.0	1.5	1.8	1.9
**6**	5.2	3.3	2.3	2.3
**7**	4.6	2.6	2.3	2.5
**8**	2.9	2.0	1.6	1.5
**9**	5.7	3.0	3.5	3.5
**10**	9.2	1.5	1.5	1.5
**11**	12.9	6.2	7.2	7.2
**12**	3.2	2.0	1.9	1.9
**13**	4.3	2.6	2.5	2.4
**14**	6.3	3.1	2.5	2.4
**15**	4.2	1.7	2.1	2.1
**16**	5.9	3.2	3.6	3.6
**17**	1.5	1.5	1.1	1.1
**18**	5.0	2.4	1.9	2.1
**19**	1.9	0.9	1.0	1.0
**20**	5.2	1.0	1.0	1.1

Abbreviations: IPSA inverse planning simulated annealing, HIPO hybrid inverse planning and optimization, CTV clinical target volume, OAR organ at risk, Man mandible, Ton tongue

The number of active positions per catheter are shown in Table 5. The mean numbers of active positions were 3.6, 5.2, 5.4, and 5.5 for IPSA, HIPO_CTV, HIPO_OAR_Ton, and HIPO_OAR_Man, respectively. The number of active positions of IPSA was lower than those of the HIPO plan for most patients. The difference between the IPSA and HIPO_CTV was statistically significant (*p* < 0.001). The SD of the number of active positions per catheter of IPSA was larger than that of the HIPO plans.

**Table 5:** Number of active positions per catheter for each plan.

Patient number	Number of active positions per catheter (± standard deviation of the number of active positions per catheter channel)			
	IPSA	HIPO_CTV	HIPO_OAR_Ton	HIPO_OAR_Man
**1**	1.4 ± 0.9	4.6 ± 1.5	5.0 ± 1.8	5.0 ± 1.8
**2**	2.8 ± 1.0	5.4 ± 0.5	4.8 ± 0.7	5.4 ± 0.5
**3**	4.8 ± 0.9	5.3 ± 1.4	6.0 ± 1.2	6.0 ± 1.2
**4**	4.8 ± 1.5	8.3 ± 0.8	7.3 ± 0.4	7.8 ± 0.4
**5**	9.5 ± 2.2	11.3 ± 2.1	11.3 ± 2.1	11.0 ± 2.0
**6**	5.0 ± 1.0	4.2 ± 0.9	5.5 ± 0.5	5.8 ± 0.7
**7**	2.7 ± 1.2	4.2 ± 0.9	4.5 ± 0.8	4.5 ± 0.8
**8**	4.5 ± 1.0	4.7 ± 0.5	5.0 ± 0.8	5.0 ± 0.8
**9**	5.4 ± 0.8	5.2 ± 0.4	5.2 ± 0.7	5.2 ± 0.7
**10**	1.8 ± 0.9	4.0 ± 0.0	4.0 ± 0.0	4.0 ± 0.0
**11**	5.3 ± 1.5	7.5 ± 0.5	7.8 ± 1.1	7.8 ± 1.1
**12**	1.8 ± 1.2	3.8 ± 0.4	3.5 ± 0.7	3.5 ± 0.7
**13**	3.8 ± 0.4	6.2 ± 0.7	6.2 ± 0.4	6.4 ± 0.5
**14**	2.6 ± 1.6	4.4 ± 0.9	4.0 ± 0.7	4.1 ± 0.8
**15**	3.2 ± 1.2	5.2 ± 0.7	5.4 ± 0.8	5.6 ± 0.5
**16**	1.9 ± 0.6	4.3 ± 0.9	4.3 ± 0.7	4.3 ± 0.7
**17**	4.0 ± 0.8	4.0 ± 0.8	5.7 ± 0.7	5.7 ± 0.7
**18**	2.7 ± 1.2	4.4 ± 0.7	4.1 ± 0.6	4.4 ± 0.9
**19**	2.8 ± 0.4	5.0 ± 0.0	5.0 ± 0.0	5.0 ± 0.0
**20**	1.9 ± 0.7	4.0 ± 0.8	4.1 ± 0.7	3.9 ± 1.0

Abbreviations: IPSA inverse planning simulated annealing, HIPO hybrid inverse planning and optimization, CTV clinical target volume, OAR organ at risk, Man mandible, Ton tongue

**Fig 1:**
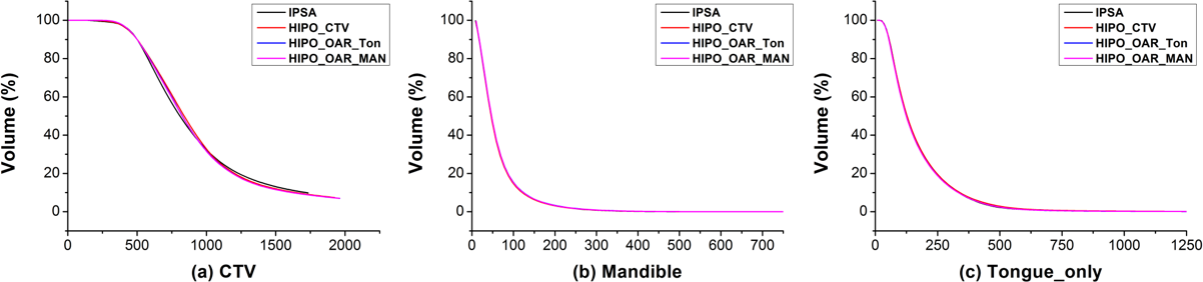
Average dose-volume histograms (DVHs) of the 20 study patients. (a) CTV, (b) Mandible, (c) Tongue_Only. IPSA, inverse planning simulated annealing; HIPO, hybrid inverse planning and optimization; CTV, clinical target volume; OAR, organ at risk.

**Fig 2:**
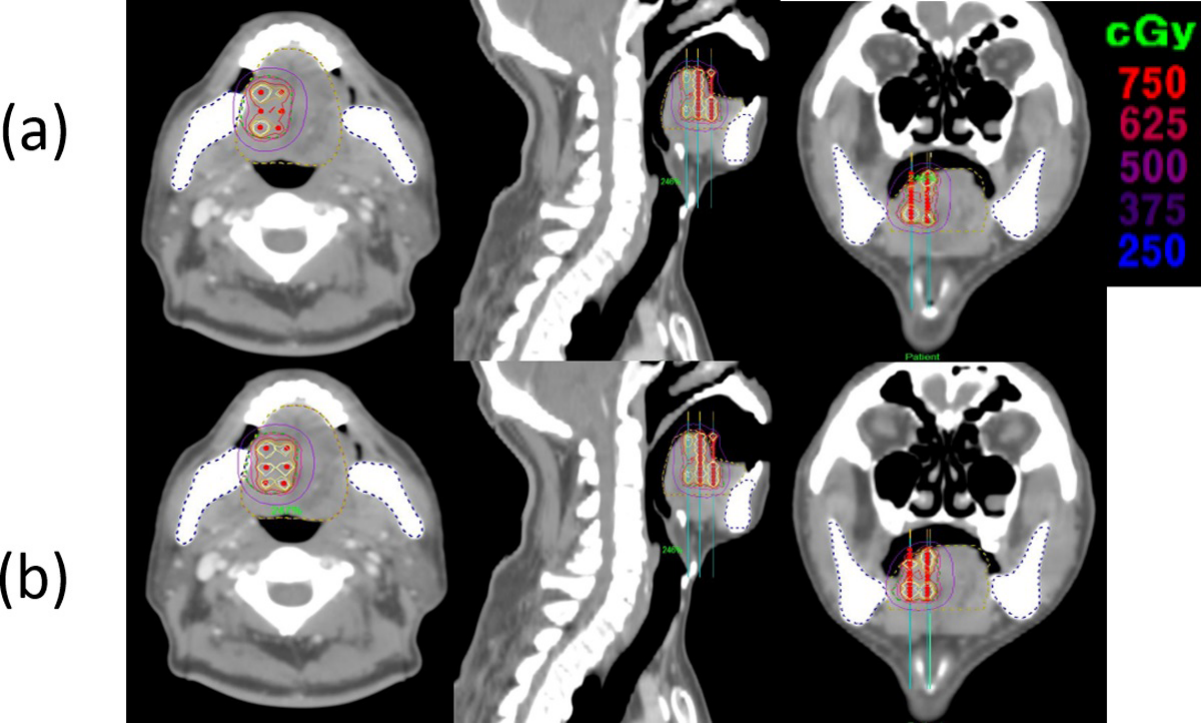
**Representative case (patient 5) showing the calculated dose distributions in the axial, coronal, and sagittal views of the (a) IPSA and (b) HIPO_CTV plans.** IPSA, inverse planning simulated annealing; HIPO, hybrid inverse planning and optimization; CTV, clinical target volume; OAR, organ at risk; Man, mandible; Ton, tongue.

Fig 1 shows the mean dose-volume histograms for the CTV, Mandible, and Tongue_Only for the 20 study patients. Fig 2 illustrates the calculated dose distributions in the axial, coronal, and sagittal views of the IPSA and HIPO_CTV plans.

The clinically relevant dosimetric parameters obtained for the planning target volume and OARs are presented in Table 6. Regarding the dose-volumetric parameters related to the CTV, V_150%_(%) of IPSA were slightly lower than those of HIPO with statistical significance (*p* < 0.001) while V_200%_(%) of IPSA were slightly lower (within SD) than those of HIPO with no statistical significance (*p* = 0.232). The three HIPO plans showed similar values. For the dose-volumetric parameters related to normal tissue, those of IPSA also tended to be similar with those of HIPO. However, the differences were not statistically significant.

**Table 6:** Dose volumetric and planning parameters of each plan.

Parameters		IPSA	HIPO_CTV	HIPO_OAR_Ton	HIPO_OAR_Man	*p*	
						IPSA/HIPO_CTV	HIPO_plans
**Target volume**							
CTV	D_95%_ (Gy)	452.9 ± 10.1	446.0 ± 12.1	445.5 ± 13.6	445.4 ±13.5	0.031	0.987
	V_150%_ (%)	56.2 ± 4.8	61.4 ± 5	60.7 ± 4.7	60.6 ± 4.9	0.001	0.859
	V_200%_ (%)	31.1± 5.8	33.9 ± 8.5	33.7 ± 0.2	33.7 ± 9.3	0.232	0.997
	Conformity index	1.17 ± 0.13	1.23±0.20	1.23 ± 0.19	1.23 ± 0.19	0.024	0.065
**Organs at risk**							
Mandible	V_2Gy_ (ml)	2.06 ± 1.16	2.03 ± 1.29	2.17 ± 1.47	2.15 ± 1.40	0.419	0.936
Tongue _only	Mean (Gy)	126 ± 28.9	127.6 ± 28.2	126.2 ± 27.9	125.9 ± 28.1	0.857	0.978

Abbreviations: IPSA inverse planning simulated annealing, HIPO hybrid inverse planning and optimization, CTV clinical target volume, OAR organ at risk, Man mandible, Ton tongue

**Fig 3:**
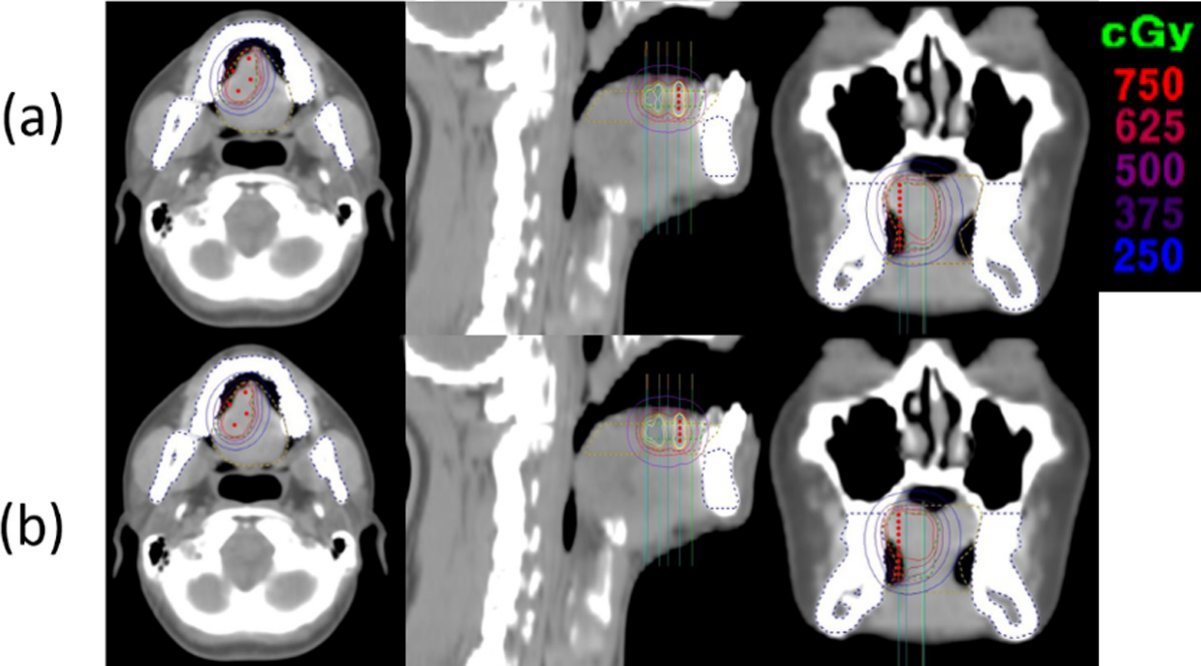
**Representative case (patient 8) showing the edited dose distributions by GrO in the axial, coronal, and sagittal views of the (a) before and (b) after GrO for HIPO_CTV plans.** Graphical optimization; (GrO), inverse planning simulated annealing; HIPO, hybrid inverse planning and optimization; CTV, clinical target volume; OAR, organ at risk; Man, mandible; Ton, tongue.

The only HIPO plans of eight patients was not achieved to clinical criteria of CTV coverage. GrO was performed for these patients. The isodose line change was shown in Fig 3. After GrO, CTV coverage was within 90–91%. The total dwell time was 13 ± 3%, 16 ± 2% and 17 ± 3% increase compared to normalization plan for HIPO_CTV, HIPO_OAR_Ton and HIPO_OAR_Man, respectively. The V2Gy of mandible was 0.9 ± 0.5%, 0.8 ± 1.1% and 2.4 ± 1.9% decrease compared to normalization plan for HIPO_CTV, HIPO_OAR_Ton and HIPO_OAR_Man, respectively.

## Discussion

In this study, the dose-volumetric parameters of target and OAR of ISPA plans were little lower than those of HIPO plans. The difference of V_150%_(%) related to target shows statistical significance, while V_200%_(%) and those of the OAR couldn’t reach statistical significance. This means that IPSA and HIPO algorithm could generate similar treatment plans for tongue cancer in terms of dose-volumetric parameters. However, total dwell time of ISPA plans was longer than that of HIPO. The dwell position distributions of IPSA were less homogeneous than those of HIPO.

The number of catheters is determined by the cross-sectional area of the CTV, and the number of active positions per catheter is determined by the thickness of the CTV. In the case of tongue cancer, the target volume is relatively small. Especially, the target thickness is frequently less than 2 cm, limiting the number of active positions per catheter. Moreover, the total dwell time depends strongly on the total number of active dwell positions. There is a correlation between the number of catheters and the total dwell time. A large number of catheters relates to an increased number of possible dwell positions, and a longer source movement time may thus be needed. However, the source movement velocity is very fast compared with the dwell time and can therefore be ignored [30].

In the present study, the prescription dose was the same for both HIPO and IPSA. As the source should stay long at each active position, the dwell time at each active position of IPSA was much longer than that of HIPO. As a result, the total dwell time of the IPSA plan was longer than that of the HIPO plans. The three HIPO plans showed similar total dwell times, owing to similar numbers of active positions per catheter.

In terms of the number of active positions per catheter, IPSA associates with fewer positions and more heterogeneous dwell times than HIPO. In particular, in each catheter, the dwell positions that are located at both ends of an activated point have long dwell times [17]. In contrast, the dwell times are very short or zero at other points between the end points [27]. These dwell time distributions can generate local hot spots at the CTV border. Moreover, an underdose region inside the CTV and overdose points in the OARs can be caused by setup errors due to catheter offset. On the other hand, HIPO shows more homogeneous dwell times and position distributions, reducing the potential risk associated with displacement of the catheters.

The clinically relevant dose-volumetric parameters were analyzed for the CTV, mandible, and tougue_only. As a result, the V_150%_ and V_200%_ to the CTV obtained with HIPO plan were found to be slightly higher than those with IPSA plan. The mean differences were in the order of 2–5%. The clinical tolerances were satisfied for all patients.

Similarly, the V_2Gy_ (ml) to the mandible and mean dose (cGy) of the tongue_only of HIPO tended to be higher or similar than those of IPSA. However, there was no statistical significance. During optimization processing, the dosimetric constraints were determine to cover the prescription to the 90% of CTV. Anshuma *et al*. reported that the maximum dose limit to the mandible was lower than 2 Gy/ml without lead shielding. Although the HDR technique allows a high dose gradient region, because the CTV was adjacent to the mandible, it was impossible to cover the CTV while optimizing the dose limit to the mandible in the present study. To prevent late complications (e.g. mandibular osteonecrosis), a spacer or lead shielding should be used during the treatment [3].

At the dwell positions along the CTV boundary, the dwell times of IPSA were longer than those of HIPO. Further, the plans calculated with HIPO showed lower minimum doses than those obtained with IPSA. The CI was calculated to evaluate the conformity for each plan using IPSA or HIPO optimization. As the CTV of tongue cancer has an irregular shape and small volume, the CI value is relatively large compared to that for other radiation therapy techniques (e.g. 3D-conformal radiotherapy or intensity-modulated radiotherapy). The calculated CI values showed that the IPSA plans can generate slightly better conformity to the target volume than the HIPO plans, regardless of the target size. This tendency resulted in IPSA being more conformal to the CTV and in a reduced dose to the OARs, as compared to HIPO.

On the other hand, there were no differences in the dosimetric plan parameters and dwell times between the three HIPO plans, which had the same dose volume constrains but different priorities. In the process of optimization, the conflicted point among the objectives was small. None of the regions of interests showed any overlapping area with other structures, and the constraints were set to satisfy the target coverage.

In this study, all plans were generated by IPSA or HIPO algorithms. After optimization, normalization were performed to improve the plan quality. GrO was performed to satisfy CTV coverage for 8 patients which cannot achieve the goal of coverage. If small modifications are performed for the first generated plan, the quality of the plan can be improved; such small modifications are usually performed by GrO [21], which is commonly used for plane optimization for HDR. However, multiple drag-and-drop actions must be applied to adjust the isodose lines by a dosimetrist. This work is time-consuming and labor intensive, taking up to 2 hours when performed by experienced users. However, if the plan generated by IPSA or HIPO optimization is modified by GrO, the process requires less than 30 minutes, including IPSA or HIPO optimization [19].

## Conclusions

For the 20 tongue cancer patients included in the present study, treatment plans could be obtained with IPSA and HIPO. In the case of HIPO, three plans were generated by assigning different priorities. These three plans were comparable in terms of the dose volume parameters, dwell time, and the dwell position and dwell time distributions. Conversely, HIPO was able to create similar treatment plans than IPSA in consideration of the plan dose distribution. However total dwell time of HIPO is shorter than that of ISPA. In term of treatment time, HIPO has an advantage.

## Supporting information

S1 File Data of planning. The results included the dwell position, time and DVH data.
(XLSX)
